# A Case Report on Hypokalemic Periodic Paralysis

**DOI:** 10.7759/cureus.72652

**Published:** 2024-10-29

**Authors:** Riana Kumarajothy, Anwar Ul-Haq, Muhammad M Akhtar

**Affiliations:** 1 Specialty and Integrated Medicine, Leeds Teaching Hospitals NHS Trust, Leeds, GBR; 2 Acute Medicine, United Lincolnshire Hospitals NHS Trust, Lincoln, GBR; 3 General Medicine, Nottingham University Hospitals NHS Trust, Nottingham, GBR

**Keywords:** flaccid weakness, hypokalaemia, hypokalemic periodic paralysis (hypopp), infection, stress

## Abstract

Low serum potassium levels can precipitate a rare condition called hypokalemic periodic paralysis. A patient may be predisposed to this phenomenon through hereditary or acquired causes. We present a case of a 21-year-old female who presented with sudden-onset generalized weakness and hypokalemia, which was treated promptly in the Accident and Emergency department, which led to a quick resolution of symptoms. This case highlights the importance of prompt diagnosis and treatment at the time of presentation to prevent the progression of symptoms and further complications.

## Introduction

Hypokalemic periodic paralysis is a rare phenomenon in which one experiences a sudden weakness in all limbs, which occurs simultaneously with low potassium levels. The paralysis typically self-resolves but can last several hours [1]. The cause can be hereditary, whereby there is a mutation in either the calcium or sodium ion channel gene or acquired such as renal or gastrointestinal losses through renal tubular acidosis or gastroenteritis [1]. Other acquired causes include thyrotoxicosis, which is when there are high levels of thyroid hormones present, with the most common cause being Grave’s disease [2]. Most familial cases are related to the CACNA1S mutation, which affects calcium channels. Typically, patients who experience hypokalemic periodic paralysis tend to have more than one attack, which varies from weekly to monthly, especially when it is familial in nature. There are also triggers to such episodes such as infection, strenuous exercise, a high carbohydrate meal, or high salt intake [3]. Frequency and severity can be controlled with low-dose potassium supplements and potassium-sparing diuretics [3].

In this case report, we will discuss a presentation of hypokalemic periodic paralysis to the local Accident and Emergency department.

## Case presentation

A 21-year-old Caucasian female presented to the Accident and Emergency Department with sudden-onset generalized weakness. Along with this, she also had a worsening sore throat that had been ongoing for three days, which was not associated with fevers or any other flu-like symptoms. She was alert and orientated to time, place, and person, and her speech was normal. Her Glasgow Coma Scale (GCS) was 15/15, and her pupils were equal and reactive to light bilaterally. On inspection, there was no obvious facial asymmetry and no obvious muscle wasting in her limbs. Cranial nerves II-XII were intact, and the deep tendon reflexes appeared reduced. Her power on examination displayed flickers or minimal contraction throughout all limbs, including proximal and distal muscles, in keeping with a power of 1/5, though sensation was intact. Vibration and proprioception were not tested at this time. There was no reported trauma preceding these symptoms. She did not have any previous medical history though this was not her first presentation for a similar episode, and she has suffered with bouts of generalized weakness and low potassium since the age of 15 years. The patient did not disclose any known family history of kidney disease, genetic disorders, or similar presentations in her first-degree relatives. She was prescribed Acetazolamide 250 mg twice a day as prophylaxis for hypokalemic periodic paralysis by her GP. 

Her vitals on admission and throughout were stable. An electrocardiogram was done, which did not have any features of hypokalemia or arrhythmia. A venous blood gas was taken on attendance, and the results are shown in Table 1. The blood results taken on admission are shown in Table 2.

**Table 1:** Venous blood gas on admission

Parameter	Result	Reference Range
pH	7.375	7.320-7.430
Sodium (mmol/L)	142	133-146
Potassium (mmol/L)	2.1	3.5-5.3
Chloride (mmol/L)	105	95-108
Lactate (mmol/L)	0.8	0.5-2.2
Actual Bicarbonate (mmol/L)	27.3	22.0-29.0

**Table 2:** Blood results on admission GFR: glomerular filtration rate

Test Name	Result	Reference Range
Hemoglobin (g/L)	124	117-149
White Cell Count (10*9/L)	15.9	4.3-11.2
Platelets (10*9/L)	265	150-400
Neutrophils (10*9/)	13.16	2.1-7.4
Sodium (mmol/L)	141	133-146
Potassium (mmol/L)	2.1	3.5-5.3
Urea (mmol/L)	2.5	2.5-7.8
Creatinine (umol/L)	49	45-84
GFR (ml/min)	>90	90-200
Adjusted Calcium (mmol/L)	2.43	2.20-2.60

Blood workups on attendance were in keeping with a possible infection and a severely low potassium level. Tying in clinical history and examination with biochemical results, the working diagnosis was hypokalemic periodic paralysis secondary to an acute sore throat.

Treatment for hypokalemia was immediately initiated with two bags of sodium chloride 0.9% with 40 mmol of potassium chloride in each bag. Alongside this, oral potassium supplementation was also initiated.

Following this treatment, power was regained, and examination showed power described as active movement against gravity and resistance throughout all limbs, in keeping with a power of 4/5. The symptoms resolved in less than 20 hours following prompt treatment. Potassium levels following treatment had improved to 4.9 mmol/L. She was reviewed and discharged with oral potassium supplementation and continued Phenoxymethylpenicillin as prescribed by her GP for an acute sore throat. She was also advised on lifestyle measures to reduce the recurrence. She has not since had any further attendance at the hospital with this presentation.

## Discussion

In this case, the cause of her symptoms was quite clear, given that she had previously presented similarly and other potential causes were unlikely. It is possible that oral intake was reduced during the course of the sore throat, hence the possibility of low potassium occurring and the patient being more susceptible to this phenomenon given her previous episodes. However, the patient has not previously seen a specialist and the cause that predisposes the patient to this phenomenon remains unknown.

Hypokalemic periodic paralysis, as outlined in the introduction, is a rare phenomenon that can be caused by acquired and hereditary causes. Acquired causes include thyrotoxicosis and renal and gastrointestinal losses [1]. In the event of thyrotoxicosis, which was unlikely in this patient’s presentation given the unremarkable thyroid function, high thyroid hormones can lead to low serum potassium through the stimulation of the beta 2 adrenergic receptors, resulting in an increase in the activity of the sodium-potassium pump [2]. Treatment is with prompt correction of potassium and thyroid hormone levels [2].

On the other hand, hereditary causes are due to mutations in the *CACNA1S* and *SCNA4A* genes and are both of autosomal dominant inheritance [4]. *CACNA1S* is a gene coding for a voltage-dependent L-type calcium channel that plays a role in the excitation-contraction coupling and the *SCN4A* gene encodes a subunit of the voltage-gated sodium channel, both working in the skeletal muscles [5]. Skeletal muscles are the area that holds the largest amount of potassium ions [2] and when an individual has a preexisting mutation disrupting the flow of current through the channels, low serum potassium levels can exacerbate this and disrupt the action potential, leading to paralysis [5].

The symptoms of paralysis can be typically prevented with prophylaxis acetazolamide and oral potassium supplementation unless a trigger occurs, such as stress through infection or strenuous exercise, or a high carbohydrate meal [1]. Acetazolamide is a carbonic anhydrase inhibitor that works by inducing metabolic acidosis in the kidneys leading to the movement of potassium and increased serum levels [6]. It is important to keep in mind that acetazolamide may worsen symptoms in patients with a mutation in the *SCN4A* gene, which causes sodium channel inactivation [7]. In the event that carbonic anhydrase inhibitors are not suitable, potassium-sparing diuretics can also be used [7]. In this case, the patient had been on acetazolamide for the last few years to reduce the frequency of attacks. In addition, it is also important to counsel the patient on triggers of hypokalemia periodic paralysis in order to further reduce the recurrence as well as the length of attacks.

## Conclusions

Hypokalemic periodic paralysis is rare but should be recognized and treated promptly as is in this case, to avoid prolongation of symptoms and to prevent further episodes. It is also important to investigate the cause of such a phenomenon in an individual and to ensure prompt electrocardiogram monitoring if clinically indicated. Following prompt treatment, it is also important to advise lifestyle changes such as reduced salt intake and avoidance of demanding exercise and carbohydrate load diet.
